# Self-Efficacy and Clinical Characteristics in Casual Gamers Compared to Excessive Gaming Users and Non-Gamers in Young Adults

**DOI:** 10.3390/jcm9092720

**Published:** 2020-08-22

**Authors:** Sun Ju Chung, Joon Hwan Jang, Ji Yoon Lee, Aruem Choi, Bo Mi Kim, Min Kyung Park, Myung Hun Jung, Jung-Seok Choi

**Affiliations:** 1 Department of Psychiatry, SMG-SNU Boramae Medical Center, Seoul 07061, Korea; sunjujung1991@gmail.com (S.J.C.); idiyuni91@gmail.com (J.Y.L.); choiar90@gmail.com (A.C.); dreamykim@gmail.com (B.M.K.); reneedrv@gmail.com (M.K.P.); 2 Department of Psychiatry, Seoul National University Health Service Center, Seoul 08826, Korea; jhjang602@naver.com; 3 Department of Medicine, Seoul National University College of Medicine, Seoul 03080, Korea; 4 Department of Psychiatry, Hallym University Sacred Heart Hospital, Hallym University College of Medicine, Anyang 14068, Korea; mhjung@hallym.or.kr; 5 Department of Psychiatry and Behavioral Science, Seoul National University College of Medicine, Seoul 03080, Korea

**Keywords:** internet gaming disorder, game addiction, casual gamer, self-efficacy

## Abstract

This study investigated differences in the self-efficacy and clinical characteristics which were found relevant to addictive behaviors in young adults according to time spent gaming. To our knowledge, this is the first study to explore self-efficacy in casual gamers relative to patients with internet gaming disorder (IGD) and non-gamers. In total, 158 young adults participated in this study and were divided into three groups: excessive gamers, who were diagnosed with IGD based on the Diagnostic and Statistical Manual of Mental Disorder-fifth edition (DSM-5, *n* = 71); casual gamers, who played games regularly but did not meet the criteria for IGD (*n* = 37); and non-gamers/controls, who did not engage in gaming (*n* = 50). All participants completed self-administered questionnaires, including measures of self-efficacy and clinical features such as the Barratt Impulsiveness Scale, Beck Depression Inventory, Beck Anxiety Inventory, Behavioral Activation/Inhibition Systems, aggression, and psychosocial well-being. There were significant differences in the self-efficacy according to the extent of gaming (excessive gamers < casual gamers < non-gamers). In addition, aggression, impulsivity, depression, anxiety, level of stress, and behavioral inhibition system scores were significantly higher in excessive gamers than in casual gamers and non-gamers. These findings showed that individuals who spend more time playing games tend to have lower self-efficacy. Our study suggests that self-efficacy may protect against or constitute a risk of excessive gaming, particularly among casual gamers. It is necessary to pay attention to enhancing psychological well-being through self-efficacy to prevent addiction in young adult gamers.

## 1. Introduction

Internet gaming addiction has recently emerged as a major social issue, as excessive gaming interferes with daily functioning, including in several psychosocial domains [1,2,3]. Indeed, previous studies have found that various clinical impairments are related to pathological gaming. For example, individuals who excessively engage in internet gaming tend to have lower well-being [4], problems with real-life functioning (e.g., school, work, relationships, social inhibition) [5,6,7,8,9,10,11,12,13], depression [12], hostility and aggression [14,15], stress [5], loneliness [4], lower self-esteem [16], and cognitive dysfunctions including problems with decision making [17], attention [5,18], and memory [19]. In addition, problematic gamers have higher rates of sleep problems and irritability and weaker self-regulation or mood stability compared to non-problematic users [20,21].

The gaming population also includes individuals who play regularly but are not addicted; these people are called “casual gamers” or “regular gamers”. The global rates of game use are growing [1,22], and risk of gaming addiction increases as time spent playing increases [23]. Given that time spent gaming is a significant risk factor for internet gaming disorder (IGD) [24], it is not surprising that some casual gamers become addicts. Indeed, a few studies have investigated the characteristics of this group. For example, casual players report weaker neuroticism compared to gaming addicts and non-gamers [25]. In terms of cognitive functioning, Metcalf and Pammer [26] studied players of first-person shooter (FPS) games and found that casual gamers performed better on a decision-making task compared to controls [26]. Electroencephalographic data have revealed significant differences between excessive and causal gamers, suggesting that excessive gamers show increased emotional processing in response to cues related to computer games compared to casual gamers [27]. However, these findings do not provide detailed information about casual gamers in that most of these findings focused on the characteristics of gaming addicts compared with casual players, and it is hard to conclude whether there are any common characteristics which could pose a risk for gaming addiction in casual gamers. In this context, it is difficult to draw consistent conclusions about such gamers. Therefore, we need to ascertain the characteristics of casual gamers versus excessive gamers and non-gamers to prevent or delay the development of IGD.

Self-efficacy is defined as one’s belief or confidence in one’s ability to cope with demands in a variety of contexts [28]. Individuals with stronger self-efficacy are more likely to meet goals or devote effort to a task [29,30,31]. This concept has been used in several addiction studies, as self-efficacy may be associated with many addictive behaviors, such as alcohol use, substance abuse, smoking, and problematic eating behavior [32,33,34,35,36], and it could operate differently in various domains, which is applicable to a specific situation or covers a wide range such as general self-efficacy. Especially, it was found that self-efficacy was relevant to internet addiction and game use. For example, some previous studies revealed that internet self-efficacy, which is involved in the regulation of one’s internet usage, is among the significant predictors of problematic internet use [37,38]. Odacı (2013) also found that self-efficacy in an academic setting was a significant predictor of excessive internet use [39]. Another study suggested that refusal self-efficacy, which refers to the ability to refuse or resist participation in substance use behavior, is negatively associated with internet addiction [40]. In respect to game use, there was a tendency for gaming addicts to have lower general self-efficacy in various age groups [41]. Similarly, social self-efficacy, which involves conviction for one’s self in maintaining or developing social relationships, may be negatively related to gaming addiction in real space but positively associated with it in virtual space [42]. 

Considering the abovementioned findings, it can be inferred that one’s degree of self-efficacy may be related to the extent of game use, where excessive gamers have much weaker self-efficacy. However, there remains a lack of empirical support for the potential associations between game use and self-efficacy due to a shortage of comparison groups or the limitation of range for self-efficacy previously investigated. In addition, it is important to investigate how it may vary depending on gaming levels, as non-addicted gamers may differ from addicted gamers and so forth.

Therefore, we investigated differences in self-efficacy according to the extent of internet gaming. We used a general self-efficacy scale, which measures perceived competence in a broad and consistent context. Because the present study includes non-gamers who do not play any internet games, there is a need to figure out self-efficacy focusing on a general dimension rather than paying attention to specific situational self-efficacy. 

Furthermore, we also tried to identify the clinical characteristics associated with IGD. As mentioned above, there are several clinical variables which have already been found to be associated with IGD in preceding studies such as impulsivity [24], Behavioral Inhibition/Activation Systems [24], depression [12], anxiety [24], aggression [14,15], and psychological distress [5]. As it is difficult to confirm whether these variables vary based on the level of gaming use only with prior research, the present study aimed to identify this aspect. In addition, in the sense that it was also revealed that self-efficacy is connected with various psychological maladjustment including depression, anxiety, or stress [43,44], we hypothesized that self-efficacy would have negative relationships between clinical characteristics related to IGD. If the relevance is confirmed, it will further support our assumption that self-efficacy may serve as a kind of protective factor for preventing the risk of addiction. In addition to this, we investigated whether the time spent on internet and smartphone use and the level of internet addiction and smartphone addiction differed in groups because self-efficacy is found to be related to internet addiction, as mentioned, and the higher the level of internet gaming, the easier the risk of internet addiction and smartphone addiction.

Therefore, we hypothesized first that self-efficacy would differ depending on the extent of gaming and that excessive and casual gamers would show greater impairment in this domain than non-gamers. Furthermore, we expected there to be differences in clinical variables based on the level of gaming, and we also hypothesized that the excessive and casual gamers would show greater impairment in these characteristics than non-gamers. Finally, we expected that self-efficacy would be negatively associated with these variables.

## 2. Materials and Methods

### 2.1. Participants and Procedure

In total, 158 young adults participated in this study. Participants were recruited consecutively from the SMG-SNU Boramae Medical Center, Seoul, Korea. All individuals with IGD were seeking treatment for problems related to excessive internet gaming. IGD was diagnosed based on the criteria of the Diagnostic and Statistical Manual of Mental Disorders, Fifth Edition (DSM-5) by a clinically experienced psychiatrist. Casual gamers and non-gamers were recruited via an advertisement such as a leaflet in the local community and posting promotional materials on the community websites. Those who played games regularly with personal computers or smartphones but did not meet the criteria for IGD were classified as casual gamers (CG), and those who did not play internet games and had zero gaming hours based on self-reports were categorized as non-gamers (NG) and served as healthy controls. These individuals did not meet at all the criterion of IGD and no one in the group exceeded the cut-off score of the internet addiction test, while those who exceeded the cut-off on the Young’s Internet Addiction Test (Y-IAT) were all in the IGD group. Those who scored above a cut-off score on the Alcohol Use Disorders Identification Test (AUDIT-K) [45] and those with psychotic disorders or intellectual disabilities were excluded from the selection of all participants.

### 2.2. Measures

All participants completed a questionnaire seeking basic information including sex, age, and hours spent gaming using the Internet with personal computers or smartphones as well as hours spent on the Internet and using smartphones. They also completed self-report questionnaires measuring self-efficacy, internet addiction, smartphone addiction, and clinical features associated with IGD.

#### 2.2.1. DSM-5 Diagnostic Criteria for IGD

The Diagnostic criteria of IGD in DSM-5 consists of nine items assessing withdrawal, preoccupation, tolerance, unsuccessful attempts to control internet use, lack of other interests, persistent excessive use, functional impairment, deception of other people regarding game use, and escape [46]. Participants are asked to answer ‘yes’ or ‘no’ to each question; individuals who meet at least five of the criteria and report that internet gaming plays a dominant role in their daily life are diagnosed with IGD [47]. Participants with IGD in this study played online games with personal computers or smartphones.

#### 2.2.2. Y-IAT

The Y-IAT is a 20-item self-report questionnaire that assesses the degree of internet use on a five-point Likert scale [48,49]. Total scores range from 20 to 100, and higher scores reflect higher levels of problematic internet use. Individuals with scores above 70 are considered to demonstrate clinical levels of internet addiction. The Cronbach’s α coefficient of this scale was 0.961.

#### 2.2.3. Smartphone Addiction Scale (SAS)

The SAS, which was used to evaluate smartphone addiction, requires participants to answer 33 items on a six-point Likert scale. These items assess daily life disturbances attributable to addiction symptoms, level of overuse, tolerance, and withdrawal, and so on [50]. Higher scores indicate higher levels of symptoms. The Cronbach’s α coefficient of this scale was 0.970.

#### 2.2.4. General Self-Efficacy Scale (SES)

The SES is a 10-item self-report questionnaire measuring perceived levels of self-efficacy, which refers to one’s general confidence in one’s ability to cope with challenging situations [28,51]. Higher scores indicate higher levels of perceived self-efficacy. The Cronbach’s α coefficient of this scale was 0.911.

#### 2.2.5. Aggression Questionnaire (AQ)

The AQ is a 29-item instrument that assesses tendencies toward aggression, including verbal aggression, physical aggression, anger, and hostility on a five-point Likert scale [52,53]. Higher scores indicate higher levels of aggression. The Cronbach’s α coefficient of this scale was 0.934.

#### 2.2.6. Behavioral Activation System (BAS) and Behavioral Inhibition System (BIS) Scale

Sensitivity to the rewards and punishments associated with gaming was evaluated using the BAS and BIS [54], a 20-item questionnaire. The BAS subscale addresses fun-seeking, reward responsiveness, and drive. The Cronbach’s α coefficient of this scale was 0.863.

#### 2.2.7. Barratt Impulsiveness Scale-11 (BIS-11)

Impulsiveness was measured using the BIS-11, which consists of 23 items measuring impulsivity including motor impulsivity, cognitive impulsivity, and non-planning impulsivity on a four-point Likert scale [55]. The Cronbach’s α coefficient of this scale was 0.859.

#### 2.2.8. Beck Depression Inventory (BDI-II)

The BDI-II is a 21-item self-report measure designed to assess depressive symptoms experienced over the previous two weeks on a four-point Likert scale [56]. Higher scores indicate higher levels of depressive symptoms. The Cronbach’s α coefficient of this scale was 0.947.

#### 2.2.9. Beck Anxiety Inventory (BAI)

Anxiety was measured with the BAI which consists of 21 items that measure symptoms of anxiety on a scale ranging from 0 to 3 [57]. Higher scores reflect higher anxiety levels. The Cronbach’s α coefficient of this scale was 0.944.

#### 2.2.10. Psychosocial Well-Being Index (PWI)

The Psychosocial Well-being Index (PWI) consists of 45 items that assess distress in terms of physiological and psychological responses to distressing events on a four-point Likert scale [58]. Higher scores reflect higher levels of psychosocial stress [59]. The Cronbach’s α coefficient of this scale was 0.971.

### 2.3. Statistical Analyses

Comparisons of demographic characteristics relied on a chi-square test and one-way analysis of variance (ANOVA) of means followed by Bonferroni’s test for equal variances or Welch’s ANOVA followed by Dunnett’s T3 test for unequal variances. Self-efficacy and clinical characteristics were analyzed using multivariate analyses of covariance (MANCOVA) controlling for sex. We used SPSS 21.0 version (SPSS Inc., Chicago, IL, USA) for all analyses.

### 2.4. Ethical Approval

An explanation of the research was provided to all participants and informed consent was received from all participants prior to their participation. This study adhered to the principles of the Declaration of Helsinki and was approved by the Institutional Review Board of SMG-SNU Boramae Medical Center (01-2018-26).

## 3. Results

### 3.1. Demographic Information

The demographic characteristics of the participants are presented in Table 1. In all, 71 (44.9%) of the 158 respondents met the criteria for IGD, 37 (23.4%) were casual gamers, and 50 (31.6%) were non-gamers. There were significant differences in sex (*x*^2^ = 14.632, *p* = 0.001), but not in age (F(2155) = 2.238, *p* = 0.114) among the three groups.

Those with IGD spent significantly more time on gaming on both weekdays (F(2152) = 39.062, *p* < 0.001) and weekends (F(2148) = 55.151, *p* < 0.001) compared to casual gamers and non-gamers. There were also significant differences between casual and non-gamers. Post hoc tests revealed that, compared to non-gamers, those with IGD spent more weekday and weekend hours on the Internet (F(2154) = 4.502, *p* = 0.007) and spent more weekend hours using their smartphone (F(2147) = 2.931, *p* = 0.023). However, casual gamers did not significantly differ from those with IGD in these domains. In terms of degree of internet and smartphone addiction, the IGD group showed much higher levels of addiction than either casual gamers or non-gamers in Y-IAT (F(2155) = 84.758, *p* < 0.001) and SAS (F(2154) = 28.139, *p* < 0.001).

### 3.2. Comparisons of Self-Efficacy and Clinical Characteristics

**Figure 1:**
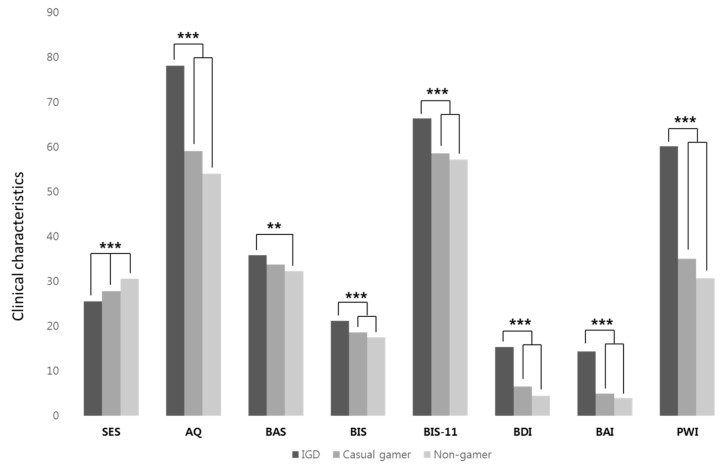
Comparison of self-efficacy and clinical characteristics adjusted for sex; IGD = internet gaming disorder; SES = Self-Efficacy Scale; AQ = Aggression Questionnaire; BAS = Behavioral Activation System; BIS = Behavioral Inhibition System; BIS-11 = Barratt Impulsiveness Scale version-11; BDI = Beck Depression Inventory; BAI = Beck Anxiety Inventory; PWI = Psychosocial Well-being Index; ** *p* < 0.01, *** *p* < 0.001.

The results of analyses of covariance controlling for sex are shown in Figure 1. The groups significantly differed in self-efficacy, which was negatively related to time spent gaming (F(2154) = 18.703, *p* < 0.001). For the IGD group, the mean SES score was 25.45 (SD = 4.95), casual gamers scored 27.78 (SD = 4.39), and the mean score of non-gamers was 30.50 (SD = 4.41).

The mean score for aggression in the IGD group was significantly higher with 78.11 points (SD = 18.53) compared to mean points of 59.00 (SD = 16.10) in casual gamers and 54.02 points (SD = 11.68) in non-gamers (F(2154) = 39.189, *p* < 0.001). For the BIS, the mean difference between the IGD group and the other groups was significant (F(2154) = 18.006, *p* < 0.001). The IGD group scored 21.13 (SD = 4.20), casual gamers scored 18.54 (SD = 3.11), and the mean BIS score in non-gamers was 17.44 (SD = 3.67). With regard to the BAS, a significant difference was observed between the IGD group (M = 35.75, SD = 5.93) and non-gamers (M = 32.18, SD = 5.15); however, casual gamers (M = 33.70, SD = 6.15) did not significantly differ from the IGD group (F(2154) = 5.429, *p* = 0.005) in this regard. We found the IGD group also scored significantly higher in impulsivity with 66.31 points (SD = 8.83) than mean points of 58.54 (SD = 10.72) in casual gamers and 57.16 points (SD = 6.96) in non-gamers (F(2154) = 20.648, *p* < 0.001). Significant differences between the IGD group and the other groups were also observed in depression (F(2153) = 25.428, *p* < 0.001) and anxiety (F(2154) = 24.623, *p* < 0.001) scales. The IGD group reported 15.26 (SD = 10.98) as the mean score of depression level, whereas casual gamers reported 6.54 points (SD = 9.63) and non-gamers reported 4.38 (SD = 4.58). In the degree of anxiety, the IGD group recorded a mean of 14.27 points (SD = 12.63), casual gamers recorded 4.87 points (SD = 6.59), and non-gamers scored 3.90 points (SD = 4.78). The level of psychophysiological distress in the IGD group was also higher with a mean score of 60.14 (SD = 26.35) compared to 35.00 points (SD = 22.20) for casual gamers and 30.64 points (SD = 16.08) for non-gamers (F(2154) = 32.275, *p* < 0.001).

### 3.3. Correlation of Self-Efficacy and Clinical Characteristics in Total Sample

In the total sample, most of the clinical variables were significantly correlated with self-efficacy. It was negatively correlated with the time spent on gaming (*r* = −0.454 *p* < 0.001; *r* = −0.433, *p* < 0.001), internet use (*r* = −0.188 *p* < 0.05; *r* = −0.280, *p* < 0.001), and smartphone use (*r* = −0.257 *p* < 0.001; *r* = −0.248, *p* < 0.001), both on weekdays and weekends. There was also a significant negative relationship between self-efficacy and internet addiction level (*r* = −0.525, *p* < 0.001), smartphone addiction level (*r* = −0.456, *p* < 0.001), aggression (*r* = −0.434, *p* < 0.001), BIS (*r* = −0.632, *p* < 0.001), impulsivity (*r* = −0.548, *p* < 0.001), depression (*r* = −0.652, *p* < 0.001), anxiety (*r* = −0.576, *p* < 0.001), and distress (*r* = −0.637, *p* < 0.001). However, the BAS was not related to self-efficacy (Table 2).

## 4. Discussion

The present study examined differences among excessive gamers, casual gamers, and non-gamers with regard to self-efficacy and clinical characteristics. To our knowledge, this is the first study to explore self-efficacy in casual gamers relative to patients with IGD and non-gamers.

Our main result is that the self-efficacy differed across groups and that casual gamers significantly differed from IGD and non-gamers in this regard; that is, the self-efficacy of casual gamers was intermediate between that of those with IGD and non-gamers. These findings suggest that individuals who engage in gaming more frequently tend to have weaker self-efficacy, which is in line with previous studies. Indeed, Festl et al. [41] found that high levels of gaming addiction are significantly associated with weaker self-efficacy among adolescents, young adults, and even older adults. Similarly, another previous study found that excessive game use was negatively related to the self-efficacy in real world situations [42]. Considering these previous studies together with the findings of our study, it could be expected that the lower the self-efficacy level the more vulnerable a person is to internet gaming addiction, and self-efficacy could be used when intervening in excessive gamers or casual gamers. Moreover, several reports that self-efficacy is associated with internet addiction have also been revealed [60,61], and these findings support our findings reflecting that self-efficacy may contribute to addictive behaviors related to internet use.

Similar findings have also been found in terms of concepts that resemble self-efficacy. Self-esteem, which is defined as one’s sense of self-worth or value and is one of the components of self-constructs along with self-efficacy, was negatively associated with internet addiction [62,63]. Additionally, several studies have suggested that self-esteem significantly predicts internet addiction [64,65], and a few studies have found that decreased self-esteem is related to online game use [66]. Working from the perspective that self-efficacy is a dimension of self-concept [67], Kim et al. [68] found that individuals diagnosed with IGD were more likely than healthy controls to have a negative self-concept with regard to both their actual and their ideal selves according to neuroimaging analyses. Similarly, the discrepancy between the actual self and the ideal self had a significant influence on the tendency to engage in pathological gaming, indicating that addicted gamers try to escape from the real world or the self via internet gaming [69,70]. Furthermore, IGD patients tend to have low levels of resilience (i.e., the ability to overcome, recover from, or adapt to significant adversity) [71,72], which is noteworthy because resilience plays a protective role in IGD [73]. Indeed, several studies have found that self-efficacy is correlated with levels of resilience, which is not surprising given that self-efficacy is considered an element of resilience [74] and that general self-efficacy is closely associated with resilience [75]. Research has also shown that perceived self-efficacy distinguishes resilient adolescents from maladaptive ones [76] and that more resilient individuals see themselves as more efficient [77,78]. In this context, this research supports that the idea that self-efficacy is associated with addiction to internet games and may be a protective or risk factor in relation to such addiction.

In addition, as expected, we found significant differences between the IGD and the other groups with regard to the variables reflecting negative psychological characteristics, such as aggression, depression, anxiety, and distress. These results are consistent with previous studies which have reported that IGD patients show higher degrees of depression [79,80], anxiety [24], aggression [15], and stress [5,80]. In other words, our findings confirm the relationship between problematic game use and psychological difficulties. We also investigated impulsivity, and the BIS/BAS, which are predictors of internet addiction or an addiction to internet games [81,82]. We confirmed that the IGD group had significantly higher levels of impulsivity, which appears to be in line with several earlier studies [80,83]. There were significant differences in the BIS scores of the IGD group and other groups, whereas there was a significant difference between only the IGD group and non-gamers with regard to BAS scores. These findings indicate that excessive gamers are markedly more sensitive to stimuli related to the removal of rewards or punishment than casual gamers or non-gamers [54]. Previous studies have reported increased BIS reactivity in individuals with internet addiction or an addiction to internet games [81,84,85]. However, in our study, the IGD group did not significantly differ from casual gamers with regard to BAS scores, which reflect a personality trait associated with reward-based addiction [86]. It is difficult to directly compare previous findings with our results as there has been no previous research on the BAS of casual gamers. Nevertheless, our findings suggest that heightened BAS sensitivity distinguishes excessive gamers from non-gamers, not from casual gamers. In addition, even if the significance of the difference between these groups had disappeared in the post hoc analyses, it would be noteworthy that casual gamers scored higher than non-gamers in almost all clinical variables measured in this study. Thus, it is likely that casual gamers are more predisposed than non-gamers to the development of internet gaming addiction and to maladaptive psychological characteristics.

This study also revealed that self-efficacy was negatively related to almost all clinical variables. These findings suggest that the lower the self-efficacy, the higher the level of maladaptive traits associated with IGD, and the more likely it is to have a predisposition to gaming addiction. However, results also showed that there was no significant relation between the BAS and self-efficacy. According to previous studies, it seems that the results on the relationship between BAS and self-efficacy are mixed. One study showed that reduced self-efficacy was related to increased levels of the BAS in high-school students with substance abuse [36]. However, Baker et al. (2017) reported BAS positively related to self-efficacy in terms of social confidence and enterprising confidence among undergraduate students [87]. It may be that the relationship between two constructs could be influenced by various factors such as the type of self-efficacy or specificity of samples, and the findings of this study also might be affected by such factors. 

This study had several limitations. First, as we used a cross-sectional design, we were unable to identify causal relationships between weak self-efficacy and addiction to internet games; longitudinal research is needed to elucidate this issue. Second, as we relied on self-reported measures, future studies should employ clinicians’ assessments. Third, the large proportion of male participants may have influenced the results.

Despite these limitations, we found a relationship between self-efficacy and addiction to internet games, and our findings expand our understanding of the characteristics of casual gamers. Our results can contribute to efforts to prevent addiction or to develop interventions for those who are at risk of becoming excessive gamers.

In summary, the present study showed that casual gamers significantly differed from patients with IGD and non-gamers with regard to self-efficacy, that is, the self-efficacy of casual gamers was intermediate between that of those with IGD and non-gamers. These findings suggest that individuals who engage in gaming more frequently tend to have weaker self-efficacy. In addition, we found significant differences between the IGD group and the casual gamers and non-gamers with regard to the variables reflecting negative psychological characteristics, such as aggression, depression, anxiety, and distress. Our findings suggest it is necessary to pay attention to enhancing psychological well-being through self-efficacy to prevent addiction in young adult gamers. Self-efficacy could be applied in various ways such as enhancing one’s confidence to abstain from or regulate engagement in addictive behaviors or coping with alternative behaviors rather than addictive behaviors in stressful situations and some provocative conditions [32]. In line with this, self-efficacy may play a role in regulation or cessation of addictive behaviors like excessive gaming and prevent increased risk of IGD.

## Figures and Tables

**Table 1:** Comparison of demographic characteristics among groups.

Variable		IGD (*n* = 71)	CG (*n* = 37)	NG (*n* = 50)	F/x^2^	*p*	Bonferroni Correction
Sex (M/%)		66 (93.0)	33 (89.2)	34 (68.0)	14.632	0.001	-
Age (years)		26.04 (6.28)	24.57 (4.29)	24.22 (3.07)	2.238	0.114 ^a^	-
Game (h)	Weekdays	4.59 (3.97)	1.73 (1.91)	0.00 (0.00)	39.062	<0.001	IGD > CG > NG
	Weekends	6.30 (4.46)	2.96 (2.33)	0.00 (0.00)	55.151	<0.001	IGD > CG > NG
Internet (h)	Weekdays	2.89 (2.33)	2.20 (1.90)	1.87 (1.03)	4.502	0.007 ^a^	IGD > NG ^b^
	Weekends	3.44 (2.88)	2.35 (2.06)	2.14 (1.21)	5.390	0.005 ^a^	IGD > NG ^b^
Smartphone (h)	Weekdays	3.98 (3.61)	3.82 (3.12)	2.77 (1.70)	2.516	0.025 ^a^	-
	Weekends	4.75 (4.02)	4.10 (3.47)	3.19 (2.01)	2.931	0.023 ^a^	IGD > NG ^b^
Y-IAT		59.82 (15.86)	34.78 (11.89)	30.90 (9.23)	84.758	<0.001 ^a^	IGD > CG, NG ^b^
SAS		98.99 (32.69)	69.81 (27.30)	62.62 (20.31)	28.139	<0.001 ^a^	IGD > CG, NG ^b^

IGD = internet gaming disorder; CG = casual gamer; NG = non-gamer; Y-IAT = Young’s Internet Addiction Test; SAS = Smartphone Addiction Scale; values are expressed as means ± standard deviations; ^a^ Welch’s ANOVA was used for unequal variance; ^b^ Dunnett’s T3 was used for unequal variances.

**Table 2:** Correlation Self-efficacy and clinical characteristics in total sample.

		1	2-1.	2-2.	3-1.	3-2.	4-1.	4-2.	5	6	7	8	9	10	11	12	13
1. SES		1															
2. Game (h)	2-1. Weekdays	−0.454 **	1														
	2-2. Weekends	−0.433 **	0.917 **	1													
3. Internet (h)	3-1. Weekdays	−0.188 *	0.342 **	0.269 **	1												
	3-2. Weekends	−0.280 **	0.324 **	0.266 **	0.784 **	1											
4. Smartphone (h)	4-1. Weekdays	−0.257 **	0.234 **	0.215 **	0.329 **	0.284 **	1										
	4-2. Weekends	−0.248 **	0.226 **	0.277 **	0.288 **	0.323 **	0.904 **	1									
5. YIAT		−0.525 **	0.589 **	0.606 **	0.359 **	0.456 **	0.198 *	0.219 **	1								
6. SAS		−0.456 **	0.359 **	0.349 **	0.343 **	0.438 **	0.388 **	0.376 **	0.683 **	1							
7. AQ		−0.434 **	0.463 **	0.458 **	0.254 **	0.299 **	0.248 **	0.214 **	0.635 **	0.588 **	1						
8. BAS		−0.075	0.283 **	0.240 **	0.224 **	0.185 *	0.162 *	0.141	0.282 **	0.329 **	0.403 **	1					
9. BIS		−0.632 **	0.393 **	0.344 **	0.161 *	0.254 **	0.203 *	0.206 *	0.555 **	0.473 **	0.530 **	0.378 **	1				
10. BIS-11		−0.548 **	0.438 **	0.428 **	0.120	0.143	0.258 **	0.262 **	0.458 **	0.384 **	0.495 **	0.280 **	0.436 **	1			
11. BDI		−0.652 **	0.542 **	0.507 **	0.276 **	0.357 **	0.288 **	0.272 **	0.627 **	0.454 **	0.691 **	0.280 **	0.618 **	.501 **	1		
12. BAI		−0.576 **	0.444 **	0.442 **	0.357 **	0.383 **	0.316 **	0.292 **	0.578 **	0.532 **	0.634 **	0.314 **	0.582 **	0.495 **	0.794 **	1	
13. PWI		−0.637 **	0.495 **	0.445 **	0.222 **	0.300 **	0.277 **	0.244 **	0.561 **	0.523 **	0.639 **	0.291 **	0.606 **	0.554 **	0.795 **	0.726 **	1
M		27.59	2.48	3.52	2.4	2.77	3.56	4.1	44.8	80.53	66.01	34.14	19.35	61.59	9.74	8.78	48.97
SD		5.12	3.46	4.2	1.94	2.35	3.03	3.42	18.93	32.51	19.46	5.92	4.13	9.73	10.32	10.63	28.42

SES = Self-Efficacy Scale; Y-IAT = Young’s Internet Addiction Test; SAS = Smartphone Addiction Scale; AQ = Aggression Questionnaire; BAS = Behavioral Activation System; BIS = Behavioral Inhibition System; BIS-11 = Barratt Impulsiveness Scale version-11; BDI = Beck Depression Inventory; BAI = Beck Anxiety Inventory; PWI = Psychosocial Well-being Index; * *p* < 0.05, ** *p* < 0.01.
